# Loneliness, boredom, and leisure in later life: associations with well-being

**DOI:** 10.1093/geroni/igag024

**Published:** 2026-03-18

**Authors:** Megan C Janke, Jaesung An, Laura L Payne, Liyuan Guo

**Affiliations:** Department of Health and Wellness Design, Indiana University Bloomington, Bloomington, Indiana, United States; Department of Hospitality, Recreation and Tourism, California State University East Bay, Hayward, California, United States; Department of Recreation, Sport and Tourism, University of Illinois at Urbana-Champaign, Urbana, Illinois, United States; College of Philosophy, Shanghai Normal University, Shanghai, China

**Keywords:** Social engagement, Meaningful activities, Affect, Social isolation

## Abstract

**Background and Objectives:**

The prevalence of both loneliness and boredom among older adults is high, and a significant association between these constructs has been identified. In this study, we investigate whether engagement in leisure activities moderates the relationship between boredom, loneliness, and emotional well-being in older adults.

**Research Design and Methods:**

We used data from the 2022 wave of the Health and Retirement Study (*N *= 551, age 50+). Leisure was assessed in terms of overall leisure repertoire (e.g., number of leisure activities), engagement frequency, and 7 types/categories of leisure. Correlations and linear regression models were used to examine associations and test for direct and moderating effects of the variables of interest.

**Results:**

Findings demonstrated that boredom and loneliness both independently and interactively predicted lower positive affect and higher negative affect, underscoring their amplifying and compensatory roles. In contrast, frequent participation in leisure activities—particularly hobbies, volunteering, and involvement in community organizations—emerged as significant predictors of positive affect, while having a larger leisure repertoire conferred limited benefit. Notably, findings suggest that engagement quality and activity frequency were more salient for well-being than the breadth of leisure interests.

**Discussion and Implications:**

The findings highlight the complex mechanisms by which leisure participation may mitigate the adverse effects of boredom and loneliness, with implications for the design of leisure education programs and community interventions targeting psychological well-being among aging populations. Promoting meaningful, regular leisure participation is recommended to enhance emotional health and reduce social isolation and loneliness in later life.

Innovation and Translational Significance: This study is one of the first to examine the interplay between leisure engagement, loneliness, and boredom and its effects on positive and negative affect in a sample of community-dwelling older adults. This study contributes to the literature in this area by examining how involvement in leisure may moderate the effects of loneliness and boredom on adults’ psychological well-being. Findings from this study have the potential to inform the development of future intervention programs effective in alleviating these public health issues.

Social isolation and loneliness are significant public health concerns among older adults. Research suggests that 34% of adults aged 50–80 feel isolated from others and 37% report a lack of companionship (National Poll on Healthy Aging, 2023). Weak social relationships in later life are associated with a 26%–29% risk in all cause premature mortality (Holt-Lunstad et al., 2015; Office of the Surgeon General, 2023) as well as increased risks of anxiety and depression (Mann et al., 2022). These statistics are even more concerning given that Americans are becoming less socially connected over time (Kannan & Veazie, 2023), and indicators such as use of technology, demographics (e.g., family size, marriage rates, living alone), and social participation suggest that these declines are likely to continue (Office of the Surgeon General, 2023).

Recent research underscores that boredom is a prevalent and critical issue among older adults and is linked with experiences of social isolation and loneliness (An et al., 2023). Studies have found a robust positive relationship between loneliness and boredom in this population, suggesting that these experiences not only frequently co-occur but may also reinforce one another, compounding risks to psychological well-being and overall quality of life (An et al., 2023). Positive affect (i.e., pleasurable moods and emotions) and negative affect (i.e., unpleasant or distressing emotions) contribute to individuals’ overall emotional and psychological well-being. Boredom is often described as a negative emotion by older adults (Hoeyberghs et al., 2018). Engaging in leisure activities is one strategy that older adults can utilize to reduce boredom and loneliness, which may contribute to their psychological well-being. Thus, this study aims to examine the associations between loneliness, boredom, and leisure among older adults and their positive and negative affect.

## Significance of boredom among older adults

Boredom may be viewed as a personal state or trait (Hunter & Eastwood, 2018), such as a response to a situation or personality characteristics, or as an activity-related emotion (van Hooft & van Hooff, 2018) that is alleviated when the activity evoking boredom is ceased. Many conceptualizations of boredom emphasize aspects of monotony and repetition; in aging research, this has widely been connected to older adults’ daily lives and routines (e.g., activities of daily living), particularly those in residential care facilities such as assisted living or skilled nursing (Gebhard & Frank, 2024). Also, boredom experiences are shaped by a confluence of constraining factors that frequently include ongoing health conditions, bereavement, altered living arrangements, and/or shifts in social roles, and diminished access to transportation or other resources (An et al., 2023). These circumstances can collectively restrict autonomy and reduce opportunities for meaningful choice, often resulting in a persistent exposure to low-stimulation environments. Importantly, such conditions not only increase the prevalence of boredom but also intensify its impact on psychological well-being, contributing to increased risks for depression, anxiety, and cognitive decline (Donovan & Blazer, 2020). Evidence also suggests that boredom in later life is closely intertwined with loneliness and social isolation, forming a reciprocal relationship where each exacerbates the other, further undermining mental health and quality of life (Losada et al., 2015). Thus, the experience of boredom among older adults extends far beyond mere monotony, constituting a significant risk factor for adverse mental health outcomes and diminished quality of life. The experience of boredom in later life may exacerbate, or be exacerbated by, social isolation and loneliness, further influencing adults’ emotional well-being.

Loneliness and boredom are often linked in the aging population (Ros, 2021), and interest in this topic has grown since the COVID-19 pandemic. However, much of what is known about the relationships between these concepts is not necessarily based on samples of older adults, and much existing data was collected during the pandemic (Hager et al., 2022; Tutzer et al., 2021) or relates to internet or smartphone usage (e.g., Orsolini et al., 2025). Existing research also tends to focus on loneliness and boredom among older adults living in residential facilities (e.g., Panthi, 2022). In addition to increasing adults’ mortality risk, boredom is significantly associated with social isolation (Hawkley et al., 2010). Some research suggests that boredom is not a consequence of inactivity—or lack of leisure—but rather loneliness (Clissett, 2001; Cohen-Mansfield et al., 2015). Studies have shown that loneliness can be caused by boredom in later life, and that older adults who believe that aging is associated with boredom are at a greater risk for loneliness (Conroy et al., 2010; Schönenberg et al., 2025). However, research simultaneously examining the concepts of leisure, loneliness, and boredom is extremely limited.

## Role of leisure engagement

Engagement in leisure activities may be critical in addressing issues of isolation, loneliness, and boredom, particularly in later life. Leisure activities are often inherently social, and leisure is important in explaining the social connectedness of older adults (Toepel, 2013). Leisure activities encourage self-determination and locus of control (An et al., 2025; Coleman & Iso-Ahola, 1993), both of which have been shown to decline among adults experiencing boredom and loneliness (e.g., Stavrova et al., 2022). Leisure activities also provide opportunities to enhance social relationships (Broughton & Payne, 2021; Chang et al., 2014), which is linked to reductions in loneliness and improved mental health among older adults (Thompson et al., 2024). Stanley and colleagues (2017) have noted that leisure education is needed to better help older adults engage in leisure and address the issue of boredom.

Boredom is a powerful indicator of whether individuals feel their engagement in activities is meaningful and are able to hold their attention (Westgate & Steidle, 2020). Although the size of one’s social network has been associated with loneliness (e.g., Schutter et al., 2022), some studies do not find that it is significantly associated with boredom (Schönenberg et al., 2025). Thus, the quality of leisure experiences may be more important than quantity—or total number of leisure activities (e.g., size of leisure repertoire)—as it relates to boredom. One qualitative study of adults in a residential facility found that participation in activities provides an opportunity to avoid monotony, and adults who engaged in self-led activities (e.g., walking, reading, knitting, gardening, or completing crosswords or puzzles) or community-led activities appeared to experience less boredom (Panthi, 2022). An and colleagues (2023) found that older adults living with chronic conditions frequently used leisure activities as self-regulatory strategies to sustain meaningful leisure engagement and a sense of purpose in their lives. Their findings indicated that engagement in leisure activities promotes social connection, helping older adults avoid loneliness and reducing their boredom. Thus, active participation in such activities not only fosters social connection and psychological resilience (i.e., the ability to adapt or recover) but also appears to play a crucial role in preventing boredom, negotiating constraints, and promoting healthy aging.

Leisure engagement has also been examined as it relates to loneliness among the aging population. Meaningful leisure has been proposed as one means of decreasing rates of social isolation and loneliness among older adults (Panthi, 2022), particularly as they transition into a new living environment (Millett et al., 2023). The types of leisure activities older adults participate in appear to be an important factor in regard to loneliness. For example, volunteering was negatively associated with loneliness in one recent study, but this was only true for individuals aged 40–64, and it did not appear to influence loneliness in people aged 65 and older (Richardson et al., 2023). However, 65+ is a large age range. Thus, there is a need to examine smaller cohorts of older adults (i.e., 65–79, and 80+). Other studies have indicated that watching TV or spending time in passive activities such as using the computer are not associated with building social connections (Toepel, 2013). Contrary to these findings, a study of Chinese older adults by Teh and Tey (2019) reported that individuals who frequently watched TV or listened to the radio were much less likely to feel lonely compared to those who did not across three waves of data. They also found that playing cards/mahjong, whether frequently or occasionally, reduced feelings of persistent loneliness but that frequent or occasional participation in social activities was only a significant predictor of less loneliness in the first two waves of their data. Thus, research suggests that types of leisure engagement and frequency of involvement may both be important factors to consider when examining the relationship between leisure, loneliness, boredom, and well-being in later life.

## Significance and purpose

Research consistently supports that leisure engagement, isolation, and boredom are all independently associated with adults’ psychological well-being, generally indicating that leisure activities positively contribute to well-being, whereas boredom and loneliness lead to reduced well-being (An et al., 2023; Dahlberg et al., 2022). However, little research has addressed our understanding of how loneliness, boredom, and leisure involvement are related among community-dwelling older adults, or the impact these constructs have on older adults’ emotional well-being. Thus, the purpose of this study was to examine the unique and combined effects of these factors in predicting positive and negative affect in later life. The present study is guided by the following research questions: (a) To what extent are boredom and loneliness associated with positive and negative affect among older adults? (b) How do leisure engagement variables (size of repertoire, frequency, and types) relate to boredom and loneliness, and do they moderate the associations between these constructs and emotional well-being? (c) Does the interaction between boredom and loneliness contribute uniquely to older adults’ emotional well-being, beyond their individual effects? and (d) Are specific types of leisure activities differently associated with positive and negative affect in the context of boredom and loneliness?

## Method

### Design and data collection

Data from the Health and Retirement Study (HRS) collected in 2022 were used in this study. The HRS is sponsored by the National Institute on Aging (NIA U01AG009740) and is conducted by the University of Michigan. The University of Michigan Institutional Review Board approved the study protocol. The HRS collects comprehensive information from individuals in middle to older adulthood, using a four-stage sampling design to obtain a representative sample of older adults in the United States while producing an oversampling of minorities (Schroeder et al., 2023). The sample for this study includes respondents who completed a face-to-face interview in 2022 and self-completed and returned the Leave Behind Questionnaire by mail (Smith et al., 2023). This data was then merged with the respondents who also completed an experimental module administered at the end of the face-to-face interview, assessing boredom, one of the main variables of interest in this study. A final sample of 551 adults had completed data on all of these measures and were included in the analyses for this study.

### Measures

The Boredom Proneness Scale–Short Form (BPS-SR; Struk et al., 2017) was used to assess boredom. This eight-item scale uses a Likert scale of 1 (strongly disagree) to 5 (strongly agree), with higher scores indicating more boredom. Example questions in this scale include “It takes more stimulation to get me going than most people” and “I find it hard to entertain myself.” This measure has been shown to have good evidence of unidimensionality and is a highly reliable and valid measure of boredom proneness (α = 0.88; Struk et al., 2017).

Loneliness was measured using 11-items from the revised UCLA Loneliness Scale (Lee & Cagle, 2017). It is assessed on a three-point Likert scale ranging from 1 (hardly ever) to 3 (often), with some items reverse-coded before an index score is averaged across all items. A higher score indicates increased perceptions of loneliness, and it has good established reliability and validity (α ranges from 0.87 to 0.89 across waves of the HRS; Smith et al., 2023).

The HRS questionnaire also collects information about the frequency of adults’ participation in activities. Individuals were asked about the frequency of their involvement in 21 activities (e.g., watch television, work on a hobby or project) on a Likert scale ranging from 1 (never) to 7 (daily). Only 20 activities were examined in this study; “care for a sick or disabled adult” is more reflective of caregiving than of leisure and thus was excluded. We assessed leisure engagement in three different ways. First, the size of one’s leisure repertoire was computed based on the total number of activities that respondents reported participating in (range 0–20). Next, the overall frequency of the adults’ leisure participation was calculated by averaging their scores across all 20 items (range 2–7) and then multiplying the mean by the size of their leisure repertoire. Finally, categories of leisure engagement were computed based on theory and prior research, resulting in seven different activity types: hobbies (bake/cook, sew/knit, do hobby), outdoor/physical activity (gardening, play sport/exercise, walk for 20 min), volunteering and participation in formal organizations (volunteer with youth, charity work, education/training, attend sport/social clubs, attend non-religious organizations, participate in community arts groups), cognitive activities (read, word games, play cards/games, writing, computer), praying (one item), spending time with grandchildren (one item), and watching TV (one item).

Emotional well-being was assessed with the Positive and Negative Affect Schedule (PANAS-X, Watson & Clarke, 1994) in this study. This measure asks respondents, “During the last 30 days, to what degree did you feel…?” and then provides 25 different feelings or emotional responses (e.g., afraid, interested, frustrated, hopeful). There are 13 items in the positive subscale and 12 items in the negative subscale, all assessed on a five-point Likert scale ranging from 1 (very much) to 5 (not at all). Scores are calculated by averaging all items across the subscale. Good psychometric properties have been reported for this scale, with α ranging from 0.89 to 0.93 across waves of the HRS (Smith et al., 2023).

Demographic and personal characteristics known to influence the variables of interest in this study were included in the analyses as covariates: gender (0 = female, 1 = male), race (0 = non-Whites, 1 = Whites), and marital status (0 = not married, 1 = married). Education was assessed as the highest degree obtained (range 0–9). Given the large age span of the sample, age was dummy-coded into categories (50–64, 65–79, and 80 years and older) and included in the analyses to better identify differences across age groups. Functional limitations were computed across 12 items assessing adults’ difficulty with fine (e.g., picking up a dime) and gross motor (e.g., climbing flight of stairs, reaching/extending arms) functioning, with higher scores indicating more limitations in functioning.

### Data analysis

Correlations and linear regression models were used to explore relationships among the study variables. IBM SPSS Statistics version 31.0 was used to run all analyses. To examine the moderating effect of key variables, interaction terms were included in the regression models (i.e., repertoire × boredom, repertoire × loneliness, frequency × boredom, frequency × loneliness, loneliness × boredom) to more comprehensively identify the influence of these constructs on psychological well-being.

## Results

The sample in this study was 43% male and 57% female, with 72% of all respondents reporting a race of White and slightly over 10% identifying as Hispanic. Approximately 52% of the sample was married, and the mean age of respondents was slightly younger than 70 (*M *= 69.89, *SD *= 9.78, range: 50–101). Twenty percent of the sample was 80 years of age or older, while 44.5% were aged 65–74, and 34.3% were between 50 and 64. Complete descriptive statistics and respondent demographic information are presented in Table 1. Correlation analyses indicated that all main constructs in this study were significantly associated, with the exception of negative affect and leisure repertoire. Table 2 presents the correlations among the main study variables.

**Table 1:** Descriptive statistics and respondent demographic characteristics.

Variable	*N*	**%**	*M*	*SD*
**Age**	545		69.89	9.78
**Male**	540	42.78		
**White**	539	71.99		
**Hispanic**	540	10.37		
**Married**	540	51.85		
**Education**	540		3.02	2.11
**Functional limitations**	551		2.19	2.71
**Leisure frequency**	551		3.27	0.77
**Leisure repertoire**	551		12.62	4.14
**Hobbies**	549		2.88	1.29
**Outdoor/physical activity**	549		4.15	1.67
**Volunteering/formal Orgs**	549		1.76	0.78
**Cognitive activities**	548		3.90	1.31
**Praying**	543		4.72	2.48
**Time with grandchildren**	542		2.96	1.88
**Watching TV**	542		6.58	1.13
**Boredom**	551		1.97	0.70
**Loneliness**	551		1.54	0.45
**Positive affect**	544		3.66	0.79
**Negative affect**	546		1.73	0.63

**Table 2:** Correlations among study variables.

Variable	Leisure frequency	Leisure repertoire	Boredom	Loneliness	Positive affect	Negative affect
**Leisure frequency**	–					
**Leisure repertoire**	.79***	–				
**Boredom**	−.38***	−.29***	–			
**Loneliness**	−.32***	−.25***	.36***	–		
**Positive affect**	.40***	.31***	−.39***	−.44***	–	
**Negative affect**	−.11**	−.03	.33***	.41***	−.42***	–
**Hobbies**			−.20***	−.12**	.27***	−.06
**Outdoor/physical activity**			−.29***	−.20***	.31***	−.14***
**Volunteer/formal Orgs**			−.24***	−.25***	.32***	−.10*
**Cognitive activities**			−.31***	−.25***	.22***	−.06
**Praying**			−.04	−.12**	.15***	−.01
**Time with grandchildren**			.06	−.11**	.08	.11**
**Watching TV**			−.02	−.02	.01	−.02

* *p* < .05.

** *p* < .01.

*** *p* < .001.

### Positive affect

**Figure 1:**
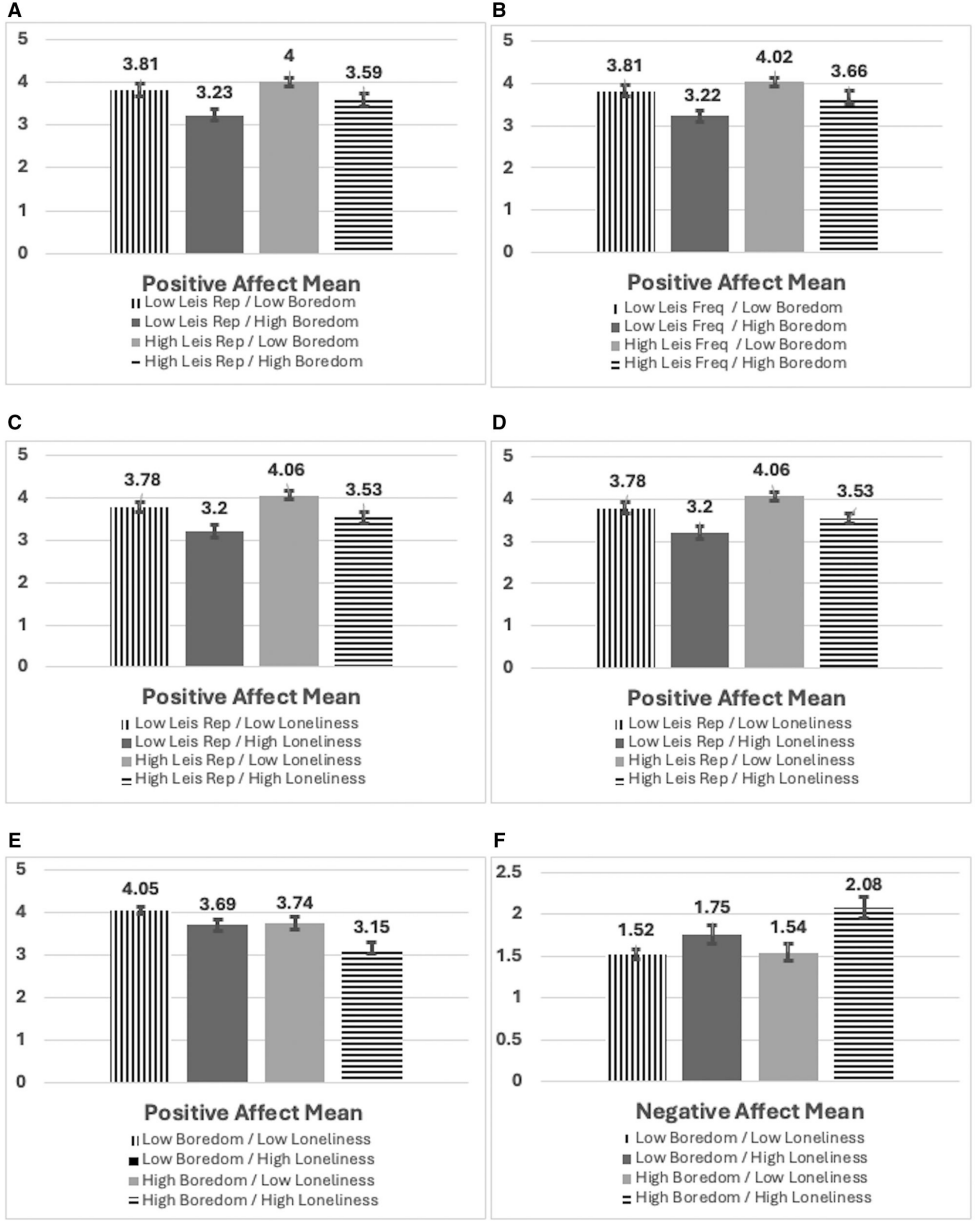
Comparison of mean values of positive and negative affect by levels of leisure, boredom, and loneliness. (A) Leisure repertoire and boredom, (B) leisure frequency and boredom, (C) leisure repertoire and loneliness, (D) leisure frequency and loneliness, (E) loneliness, boredom, and positive affect, and (F) loneliness, boredom, and negative affect.

The first regression model predicting positive affect was significant, *F*_(17,531)_ = 17.62, *p* < .001, adj *R*^2^ = .35 (Table 3), including measures of overall leisure involvement (leisure repertoire and frequency), boredom, and loneliness. Individuals who are not White (β = −0.11, *p* < .01) had lower levels of education (β = −0.09, *p* < .05) and were age 50–64 (β = −0.12, *p* < .01) had significantly lower levels of positive affect. Frequency of leisure engagement predicted positive affect (β = 0.43, *p* < .001), with individuals who engaged in leisure more frequently reporting greater positive affect. However, the size of the individuals’ leisure repertoire was not significant. Boredom proneness (β = −0.19, *p* < .001) and perceived loneliness (β = −0.29, *p* < .001) significantly predicted positive affect in the expected direction, with adults noting more boredom and less loneliness having better emotional well-being. The interaction terms included in this model were all significant in predicting older adults’ positive affect (Figure 1). The total number of leisure activities the adults reported engaging in significantly interacted with their levels of boredom (β = 0.24, *p* < .05) and loneliness (β = −0.29, *p* = .01). The frequency of adults’ participation in leisure also significantly moderated the effect of their boredom (β = −0.25, *p* < .05) and loneliness (β = 0.27, *p* < .05). For leisure repertoire, individuals with high levels of boredom had significantly lower levels of positive affect; however, those with high boredom and a high number of leisure activities in their repertoire (*M *= 4.02) reported essentially the same levels of positive affect as those with low boredom and high leisure repertoire (*M *= 4.00). For leisure frequency and boredom, adults with high boredom reported lower positive affect regardless of their frequency of engagement in leisure (*M*_LowFreq_ = 3.22; *M*_HighFreq_ = 3.66), although positive affect did increase as their frequency of leisure involvement increased, even among those with high boredom.

**Table 3:** Leisure repertoire and frequency regression models predicting positive and negative affect.

Variable	Positive affect		Negative affect	
	*SE*	β	*SE*	β
**Age 50–64**	0.06	−0.12***	0.05	0.08*
**Age 80 and older**	0.08	−0.06	0.07	−0.04
**Male**	0.06	0.05	0.05	−0.13***
**White**	0.07	−0.11**	0.05	0.05
**Hispanic**	0.09	0.04	0.08	−0.03
**Married**	0.06	0.01	0.05	0.08*
**Education**	0.02	−0.09*	0.01	−0.02
**Functional limitations**	0.01	−0.13**	0.01	0.11**
**Leisure frequency**	0.00	0.45***	0.00	−0.09
**Leisure repertoire**	0.02	−0.20	0.02	0.19
**Boredom**	0.05	−0.19***	0.04	0.21***
**Loneliness**	0.07	−0.29***	0.06	0.34***
**Leisure frequency × boredom**	0.01	−0.25*	0.00	0.13
**Leisure repertoire × boredom**	0.03	0.24*	0.02	−0.03
**Leisure frequency × loneliness**	0.01	0.27*	0.01	−0.00
**Leisure repertoire × loneliness**	0.05	−0.30**	0.04	0.02
**Loneliness × boredom**	0.10	−0.08*	0.08	0.11**
***F* statistic**		17.62***		12.32***
**Adjusted *R*^2^**		.35		.27

*Note. SE* = standard error.

* *p* < .05.

** *p* < .01.

*** *p* < .001.

For loneliness and leisure interactions, individuals with high loneliness had significantly lower levels of positive affect, but a greater size of leisure repertoire (*M *= 3.53) and leisure frequency (*M *= 3.58) increased positive affect to levels more similar to those of individuals with low levels of loneliness. The interaction between loneliness and boredom was also significant in the model (β = −0.08, *p* < .05). Individuals with low levels of loneliness (*M*_LowBored_ = 4.05; *M*_HighBored_ = 3.74) reported greater levels of positive affect, however, those with high levels of loneliness and low boredom (*M *= 3.69) reported similar levels of positive affect as adults who were low on loneliness and high on boredom (*M *= 3.74).

The second regression model predicting positive affect was significant, *F*_(19,512)_ = 14.34, *p* < .001, adj *R*^2^ = .33 (Table 4). This model included adults’ involvement in the different types of leisure activities assessed in this study. In this model, being White (β = −.10, *p* < .01) was significantly associated with higher levels of positive affect, and being aged 50 to 64 was associated with significantly lower levels of positive affect (β = −0.13, *p =* .001). Adults with greater functional limitations reported lower levels of positive affect (β = −0.10, *p* < .05). As in the above model, reports of higher perceived loneliness (β = −0.27, *p* < .001) and boredom (β = −0.21, *p* < .001) were associated with lower positive affect. The strongest leisure predictor of positive affect in this model was participation in hobbies (β = 0.13, *p <* .01), followed by volunteering and participation in formal community organizations (β = 0.11, *p <* .05), and praying (β = 0.08, *p <* .05). The other types of leisure activities were not significant predictors. The interaction between loneliness and boredom was also significant in this model (β = −0.08, *p <* .05). Individuals who reported low loneliness and low boredom had the most positive affect (*M *= 4.05), and those with high boredom and high loneliness noted the lowest levels of positive affect (*M *= 3.15). However, individuals who were very lonely but not very bored (*M *= 3.69) or very bored but not lonely (*M* = 3.74) reported similar levels of positive affect.

**Table 4:** Leisure types and leisure repertoire regression models predicting positive and negative affect.

Variable	Positive affect		Negative affect	
	*SE*	β	*SE*	β
**Age 50–64**	0.07	−0.13***	0.05	0.10*
**Age 80 and older**	0.08	−0.05	0.07	−0.01
**Male**	0.07	0.05	0.05	−0.15***
**White**	0.07	−0.10**	0.05	0.05
**Hispanic**	0.10	0.05	0.08	−0.04
**Married**	0.06	0.01	0.05	0.11**
**Education**	0.02	−0.06	0.01	−0.01
**Functional limitations**	0.01	−0.10*	0.01	0.10*
**Leisure repertoire**	0.01	0.00	0.01	0.13
**Boredom**	0.05	−0.21***	0.04	0.20***
**Loneliness**	0.07	−0.27***	0.06	0.37***
**Hobbies**	0.03	0.13**	0.02	−0.06
**Outdoor/physical activity**	0.02	0.07	0.02	−0.01
**Volunteering/formal Orgs**	0.05	0.11*	0.04	−0.02
**Cognitive activity**	0.03	−0.01	0.02	0.03
**Praying**	0.01	0.08*	0.01	−0.03
**Playing with grandchildren**	0.02	0.02	0.01	0.15***
**Watching TV**	0.03	0.04	0.02	−0.02
**Loneliness × boredom**	0.10	−0.08*	0.08	0.07
***F* statistic**		14.34***		10.85***
**Adjusted *R*^2^**		.33		.27

*Note*. *SE* = standard error.

* *p* < .05,

** *p* < .01,

*** *p* < .001.

### Negative affect

The first regression model predicting negative affect was significant, *F*_(17,533)_ = 12.32, *p* < .001, adj *R*^2^ = .27 (Table 3), including measures of overall leisure involvement (leisure repertoire and frequency), boredom, and loneliness. Female participants (β = −0.13, *p <* .001) and individuals aged 50–64 (β = .13, *p <* .05) had significantly higher levels of negative affect. Individuals reporting more functional limitations also reported higher negative affect (β = 0.11, *p <* .01). None of the leisure variables were significant in predicting adults’ negative affect. Higher levels of boredom (β = 0.21, *p <* .001) and greater perceived loneliness (β = 0.34, *p <* .001) significantly predicted more negative affect. The only interaction that significantly predicted negative affect in this model was loneliness × boredom (β = 0.11, *p <* .01). Individuals reporting low levels of loneliness reported approximately the same levels of negative affect, regardless of their levels of boredom. However, adults with high boredom and high levels of loneliness reported significantly worse negative affect (Figure 1).

The second regression model predicting negative affect included adults’ engagement in different types of leisure activities. This model was also significant, *F*_(19,514)_ = 10.85, *p* < .001, adj *R*^2^ = .27 (Table 4). Adults aged 50–64 (β = 0.10, *p <* .05), female (β = −0.15, *p <* .001), and married individuals (β = 0.10, *p <* .01), reported higher levels of negative affect. Having more functional limitations was also associated with more negative affect (β = 0.10, *p <* .05). Adults reporting higher levels of boredom (β = 0.20, *p <* .001) and loneliness (β = 0.37, *p <* .001) reported greater levels of negative affect. Only one leisure variable significantly predicted negative affect—older adults who spent more time with their grandchildren also reported greater levels of negative affect (β = 0.15, *p <* .001).

## Discussion

This study supports the connections between loneliness, boredom, leisure engagement, and emotional well-being in later life. Loneliness was a stronger predictor of positive and negative affect; however, leisure engagement was more strongly correlated with boredom proneness. These findings provide support for the qualitative study by An et al. (2023), which asserted that meaningful leisure is important to alleviating boredom. Interestingly, a larger leisure repertoire was associated with greater negative affect but not positive affect. Further investigation into the role of leisure engagement as a mediator between boredom and negative affect in later life is suggested. Findings are discussed in the context of existing literature, and we offer suggestions for future research.

### Relationships between demographics and affect

Non-White adults ages 50–64 and those with lower education levels reported lower positive affect. Health disparities shaped by social determinants of health may help explain this finding, wherein lower education may affect access to healthcare and leisure resources (Payne, 2023). Although being White was associated with higher positive affect, all adults aged 50–64 reported lower positive affect, and those aged 50–64 and women reported higher negative affect. This may be a function of membership in the “sandwich generation,” in which these adults have significant responsibilities such as caring for family members (e.g., parents, children, grandchildren) that affect emotional well-being (Lei et al., 2023).

### Leisure, boredom, and positive affect

Leisure is known to influence aspects of well-being such as positive and negative affect (Wiersma & Parry, 2010). In this study, more frequent involvement in leisure was associated with more positive affect, which is common. This finding aligns with existing research, given that leisure activities are freely chosen, intrinsically motivated, and enjoyable (Caldwell, 2005). Interestingly, leisure repertoire was not a predictor of positive or negative affect. Existing research indicates that the quality of experiences within activities represented in one’s leisure repertoire may be more important than the number of activities in one’s leisure repertoire (Lee et al., 2020), and thus, this might explain the lack of relationship to positive or negative affect in our study. Lee et al. (2020) found a curvilinear relationship between leisure repertoire and happiness, indicating that a larger leisure repertoire was associated with lower happiness. Given these findings, it appears that the size of an individual’s leisure repertoire may be less important than the meaning and value they attach to their leisure activities.

Greater boredom among participants was associated with lower positive affect. This relationship is supported by existing literature. For example, An et al.’s (2022) qualitative study found that boredom was a state that older adults actively tried to avoid due to its negative consequences on health and well-being. They articulated that higher levels of boredom contributed to feeling a lack of purpose in life, and that leisure provided structure, purpose, and meaning to life. Low arousal might also explain higher levels of boredom. Low stimulation environments contribute to low arousal, which often results in dissatisfaction (van Tilburg & Igou, 2012). However, it is harder to explain why higher boredom and increased leisure activities were associated with the same affect levels as participants with low boredom and larger leisure repertoires. This may be related to criticisms of activity theory (Havighurst & Albrecht, 1953), which asserted that more engagement in leisure activities leads to better health, when in fact, the health benefits of leisure may level off and even decline, especially with increased functional limitations. For example, a person with a wider leisure repertoire and significant functional limitations may have lower positive affect due to constraints or barriers to participation (Janke et al., 2015). In other words, there may be little use to having many activities in your repertoire if you cannot do many of the ones you love.

Although adults with higher boredom reported lower positive affect regardless of how often they engaged in leisure, frequency of leisure was associated with better affect, even among those with higher boredom. This finding indicates that adults could use leisure to improve their affect, even with higher reported levels of boredom (An et al., 2023; Lee et al., 2022). These older adults may be more self-determined to change their situation and hence utilize leisure to benefit emotional well-being. Dattilo et al. (2015) and An et al. (2025) emphasized the importance of self-determination for older adults’ ability to alleviate boredom, noting that competence, relatedness, and autonomy can motivate people to engage in leisure.

### Loneliness and leisure

In this study, adults with higher loneliness also had significantly lower positive affect. This is consistent with studies of loneliness (Davidson et al., 2022; Steptoe et al., 2011). Those with larger leisure repertoires and more frequent leisure engagement had higher positive affect, more closely reflecting positive affect levels of adults with lower loneliness scores. Given the size of their leisure repertoires, it is plausible to conclude that there are benefits to having a larger repertoire. Those with more leisure activities to choose from may be more likely to have activities that involve social engagement in their repertoires. This may increase the likelihood that they engage in activities that reduce loneliness. However, there is no guarantee that having more activities to choose from leads to choosing social activities that alleviate loneliness. In fact, Steptoe et al. (2011) found that loneliness contributed significantly to negative affect above and beyond age, gender, employment status, and depression. Negative affect also increased as the day progressed. Thus, it is important to disentangle the complexities of this relationship in future studies. Interestingly, people who reported higher loneliness scores and lower boredom also had positive affect scores, similar to those of adults with low loneliness and high boredom. This indicates there may be a bidirectional relationship between loneliness and boredom in regard to positive affect. Thus, begging the question: is positive affect a function of less loneliness despite higher boredom levels and vice versa?

The results of leisure types and positive and negative affect were consistent with previous studies. The positive relationship between aging and positive affect may be a function of people adapting to and accepting changes associated with age (Janke et al., 2015). It could also reflect more confidence in navigating aging, especially since a large percentage of participants are Baby Boomers, who are diverse and active. Also, higher functional limitations may be related to lower positive affect, which aligns with other results in this study. Moreover, Janke et al. (2015) found that leisure styles comprising sedentary activities were related to worse health outcomes than cognitive leisure styles, which aligns with the results of this study. Hobbies accounted for the most variance in positive affect. Hobbies can be lifelong activities and involve ongoing learning. The complexity and challenge associated with hobbies may contribute to positive affect. Mannell and Manske (2010) asserted that leisure is an ideal context and provides conditions necessary for complexity to develop. Hobbies might also bring people into contact with communities who share similar interests, which could also improve positive affect (Wilson & Cordier, 2013). We anticipated that volunteering and engaging with formal organizations would be associated with higher positive affect. Although not a guarantee, generally, people engage in these activities because they are meaningful and important. Prayer was also associated with positive affect, which aligns with An et al.’s (2022) study on the role of leisure in healthy aging. Some participants shared that their spiritual lives (which included prayer) were key to aging well. Although the interactions between loneliness and boredom for these activities were not significant, they approached significance and are worth examining in the future since volunteering/engaging in organizations and hobbies in particular may reduce both boredom and loneliness.

### Negative affect

Interestingly, time spent with grandchildren was the only leisure activity associated with higher negative affect. Time spent with grandchildren was asked broadly, and therefore, this activity might encompass both leisure and caregiving experiences, the latter of which might not be viewed as a leisure activity. Factors shaping negative affect for all other leisure types might be explained by factors such as age, gender, functional limitations, and potentially unmeasured factors such as depression and even fear of falling, which can contribute to negative affect. Another potential explanation is that the leisure activities measured in this study may not capture the full range of activities engaged in by older adults, which may affect these relationships. Greater loneliness and boredom predicted negative and positive affect. Research also indicates that having a positive outlook can be a protective factor for negative experiences such as loneliness and boredom (Ramos & Brown, 2020). Future research should focus on identifying mechanisms that can reduce loneliness among older adults, thereby reducing negative affect and enhancing positive affect. This is especially important given that negative affect often increases over the course of a day (Steptoe et al., 2011).

Leisure engagement was a significant predictor of positive affect but not negative affect in our models, an asymmetry that likely reflects the distinct determinants of these two components of emotional well-being. Positive affect tends to be more sensitive to situational and experiential factors, including enjoyable and meaningful leisure activities (Chen et al., 2020), whereas negative affect is more strongly governed by enduring vulnerabilities, such as personality traits, financial hardship, and mental health conditions (Asquith et al., 2022). This interpretation is consistent with research suggesting that meaningful leisure helps older adults alleviate boredom and sustain a sense of purpose and engagement, and with evidence that the quality of experiences within one’s leisure repertoire is more important for well-being than the sheer number of activities (An et al., 2023). It also aligns with studies showing that leisure and related activities explain unique variance in positive affect and flourishing, while negative affect is more strongly predicted by underlying psychological and contextual risks (Asquith et al., 2022; Choi et al., 2022). Thus, leisure may primarily *add* positive emotional experiences and build psychological resources—even among adults who report boredom and loneliness—without fully counteracting the deeper clinical or contextual drivers of negative affect.

Overall, this study offers important insights into the relationships between leisure activities, boredom, loneliness, and affect, which, when examined together, have been overlooked. However, boredom is increasingly being recognized as a significant contributor to both loneliness and affect. Thus, identifying ways to help older adults alleviate boredom may also reduce social isolation, loneliness, and negative affect. Such initiatives may include utilizing leisure education in community and supportive living settings. Leisure education increases awareness of leisure values, benefits, and resources and can increase people’s knowledge and ability to engage in meaningful and enjoyable leisure activities.

### Study limitations

There are several limitations to this study. This was a cross-sectional study that examined one wave of the HRS data. This study would be strengthened by examining these relationships over time. However, the boredom proneness scale is only currently available from one wave of HRS data, limiting the analysis. Also, the activities gathered in the HRS may not be comprehensive, and the categorization of activities, while logical, may be interpreted as somewhat subjective. There is no widespread consensus on how to classify leisure activities; thus, we drew on the existing literature and exploratory analyses to inform the leisure categories used in this study. The purpose of this research was to examine the association between different types of activities and affect, taking into account that many forms of leisure (social, cognitive, physical) have potential emotional health benefits. Therefore, in this study, all leisure activities were weighed equally. However, other means of categorizing leisure activities (e.g., physically active versus sedentary, or individual vs. group) might yield different findings among the variables. Finally, although the adjusted *R*-squares for our models were relatively high, other factors at play should be explored in future work to provide additional insight into these relationships.

## Conclusion

This study integrated boredom, loneliness, leisure participation, and emotional well-being, which is novel considering previous studies have only examined these factors independently, limiting our understanding of their effects on well-being. Results indicated that higher levels of loneliness and boredom proneness both predicted lower levels of positive affect. These findings suggest that although loneliness and boredom represent distinct psychological constructs, both showed consistently negative effects on emotional well-being, suggesting that factors such as positive outlook may be important for managing negative affect in the context of boredom and loneliness.

We also found that increased leisure participation contributed more to positive affect than just having a larger leisure repertoire. Thus, it is plausible that a smaller leisure repertoire of higher-quality activities is more important than a large leisure repertoire. In terms of leisure types, only engagement in hobbies, volunteering, and participation in formal community organizations significantly predicted higher levels of positive affect, whereas time spent with grandchildren predicted higher negative affect. In light of these findings, communities and supportive living organizations should promote leisure education and provide diverse opportunities for meaningful engagement that help to reduce boredom and loneliness, and increase emotional well-being

## Data Availability

Data, analytic methods, and materials are available to other researchers for replication purposes. Data used in this study are publicly available from the Health and Retirement Study. This study was not preregistered.
